# Modeling of Individual Fruit-Bearing Capacity of Trees Is Aimed at Optimizing Fruit Quality of *Malus* x *domestica* Borkh. ‘Gala’

**DOI:** 10.3389/fpls.2021.669909

**Published:** 2021-07-13

**Authors:** Martin Penzel, Werner B. Herppich, Cornelia Weltzien, Nikos Tsoulias, Manuela Zude-Sasse

**Affiliations:** ^1^Chair of Agromechatronics, Technische Universität Berlin, Berlin, Germany; ^2^Horticultural Engineering, Leibniz Institute for Agricultural Engineering and Bioeconomy, Potsdam, Germany

**Keywords:** apple, carbon balance, lidar, respiration, precision horticulture, growth, canopy photosynthesis model

## Abstract

The capacity of apple trees to produce fruit of a desired diameter, i.e., fruit-bearing capacity (FBC), was investigated by considering the inter-tree variability of leaf area (LA). The LA of 996 trees in a commercial apple orchard was measured by using a terrestrial two-dimensional (2D) light detection and ranging (LiDAR) laser scanner for two consecutive years. The FBC of the trees was simulated in a carbon balance model by utilizing the LiDAR-scanned total LA of the trees, seasonal records of fruit and leaf gas exchanges, fruit growth rates, and weather data. The FBC was compared to the actual fruit size measured in a sorting line on each individual tree. The variance of FBC was similar in both years, whereas each individual tree showed different FBC in both seasons as indicated in the spatially resolved data of FBC. Considering a target mean fruit diameter of 65 mm, FBC ranged from 84 to 168 fruit per tree in 2018 and from 55 to 179 fruit per tree in 2019 depending on the total LA of the trees. The simulated FBC to produce the mean harvest fruit diameter of 65 mm and the actual number of the harvested fruit >65 mm per tree were in good agreement. Fruit quality, indicated by fruit's size and soluble solids content (SSC), showed enhanced percentages of the desired fruit quality according to the seasonally total absorbed photosynthetic energy (TAPE) of the tree per fruit. To achieve a target fruit diameter and reduce the variance in SSC at harvest, the FBC should be considered in crop load management practices. However, achieving this purpose requires annual spatial monitoring of the individual FBC of trees.

## Introduction

In fruit production, the number of apples per tree is negatively correlated to the mean fruit fresh mass (FM), coloration (Palmer et al., 1997), soluble solids content (SSC) (Link, 2000; Serra et al., 2016), and flower set in the following season (Handschack and Schmidt, 1991). Each individual apple tree may initiate up to 2,000 flowers, which significantly exceeds the commercially desired number of fruit at harvest (Penzel et al., 2021). Although a high percentage of flowers and later fruitlets will be naturally shed in flower or fruit abscission, often too many fruit remain on the tree. High crop load results in low-quality fruit whereas low crop load may reduce yield. Furthermore, the distribution of fruit throughout the canopy may not be uniform, which is one reason for the variability of fruit quality within the tree. Additionally, the position of the fruit in the cluster (Jakopic et al., 2015), the position and light exposure of the bearing branch as well as the number and proximity of leaves and other fruit affect fruit quality (Belhassine et al., 2019; Reyes et al., 2020). Consequently, crop load management is required to adjust the number of fruit per tree. Various strategies to obtain one to two fruit per flower cluster widely distributed in the canopy exist, targeting a high percentage of high-quality fruit and, thus, high crop value in the current and sufficient flower bud initiation for the subsequent growing season (Costa et al., 2018). However, for developing efficient crop load management, the information on the optimal number of fruit per tree is crucial.

Much work has been done to evaluate the effects of the number of fruit per tree on apple quality parameters. ‘Gala’ apples have a high economic importance worldwide and are well described in crop load experiments. Commercial ‘Gala’ strains show a variability in mean fruit FM per tree up to 90 g affected by crop load (McArtney et al., 1996; Pilar Mata et al., 2006; Xia et al., 2009). The SSC of apples is an additional important internal quality parameter largely influencing the acceptance and buying decision of consumers. Crop load can also slightly affect the mean SSC of ‘Gala’ apples at harvest (Pilar Mata et al., 2006; Yuri et al., 2011). So far, different techniques have been applied to estimate the number of fruit per tree, which would lead to a desired fruit quality. These methods capture continuous yield recording in the orchard (Handschack and Schmidt, 1991) or the assessment of the crop load in relation to the trunk cross-sectional area (Iwanami et al., 2018). Also, the leaf area (LA) per fruit has been identified as an important determinant of fruit quality (Poll et al., 1996; Palmer et al., 1997).

Generally, trees can be considered as a collection of semiautonomous organs (DeJong, 2019), where each organ has a genetically determined, organ-specific development pattern and growth potential (Reyes et al., 2016), which is achieved according to the individual carbon supply conditions. Because only leaves perform net carbon assimilation, the exposed LA of a tree reflects the growth capacity of the tree to intercept solar radiation and serves therefore as a proxy of the fruit-bearing capacity (FBC). Lakso et al. applied LA estimates in carbon balance modeling (Lakso and Johnson, 1990). In their approach, the light interception of each individual shoot was scaled up to the canopy level by considering the tree's total LA as one big leaf, which receives the average irradiance of the canopy (De Pury and Farquhar, 1997). This approach is valuable as it combines existing knowledge in a modeling approach, providing the potential to simulate the optimum crop load. However, it may lead to an overestimation of the photosynthetic capacity of a tree because the light environments within a tree's canopy can be highly variable (Zhang et al., 2016). The photosynthesis of the exposed leaves and leaves in sun flecks is mostly light saturated whereas the photosynthetic response of shaded leaves to irradiance is linear (De Pury and Farquhar, 1997). Charles-Edwards (1982) demonstrated the validity of the big-leaf approach for hedgerow apple orchards. Furthermore, this approach was validated by recording the CO_2_ exchange of whole trees enclosed in a canopy chamber (Lakso et al., 1996). Nevertheless, the spatial variability of individual LA of trees was not taken into account in CO_2_ balance so far.

Indeed, vegetative and reproductive growths vary spatially in orchards. Variability in the trunk cross-sectional area (Manfrini et al., 2020), number of flower clusters (Vanbrabant et al., 2020; Penzel et al., 2021), yield, mean FM, and the fruit maturity stage of each individual tree (Manfrini et al., 2020) within the same orchard was described. Consequently, both the individual LA (Sanz et al., 2018) and the LA index (Poblete-Echeverría et al., 2015) and the associated FBC of each individual tree may vary spatially. It can also be assumed that such variability in each individual tree affects the optimum number of fruit per tree when targeting a homogenous fruit quality throughout the orchard. However, the actual number of fruit per tree was not yet evaluated in relation to the variable LA and associated FBC.

The mapping of canopy and yield parameters within an orchard can be performed by georeferencing each tree and the application of remote sensing, e.g., based on photogrammetry (Mu et al., 2018), time-of-flight reading (Coupel-Ledru et al., 2019; Tsoulias et al., 2019), or thermal imaging (Huang et al., 2020). Most recently, the number of flower clusters (Vanbrabant et al., 2020) and fruit per tree (Apolo-Apolo et al., 2020; Tsoulias et al., 2020a) were mapped in pome fruit orchards by analyzing the point clouds generated from RGB images or a light detection and ranging (LiDAR) analysis. The sensors may be mounted on various platforms, i.e., ground or aerial vehicles, or satellites, and the measurements carried out throughout the growth season (Zude-Sasse et al., 2016). Indeed, frequent studies of georeferencing and sensing the data of each individual tree are available, but the developed approaches lack further application in decision-support models, which can be utilized for the precise management of crop load.

Recently, the LA of each individual tree was analyzed (Penzel et al., 2020) to quantify the variability of FBC in two apple orchards. LA estimated with LiDAR compared to manual readings was obtained with high coefficient of determination (*R*^2^ = 0.96) by considering fully expanded leaves in mid-season. The authors showed that tree-adapted crop load management potentially increases the marketable yield of an orchard by 5%. Carbon balance of each individual tree would enable the adjustment of thinning intensity to each individual tree, introducing the term “variable rate application” (VRA) in crop load management. For this purpose, prototypes of precise thinning systems have been developed (Wouters, 2014; Lyons et al., 2015; Pflanz et al., 2016), but these have not been commercialized to date (Verbiest et al., 2020). This is, besides economic considerations, due to the lack of suitable models to evaluate the actual crop load of a tree in comparison to the tree's FBC.

For VRA in flower or fruit thinning, it would be advantageous to estimate FBC before full bloom or within subsequent three weeks when fruit are most susceptible to the thinning agents. For this purpose, historical data of FBC in a fully developed canopy could be analyzed, applying the previous years' data for decision-making in the current year. In viticulture, Taylor et al. (2019) proposed to utilize the crop load information from one year for crop load management decisions in the consecutive year. However, it has not yet been evaluated whether this approach can be transferred to apple production. In addition, knowledge on the effects of the absorbed light on fruit quality is lacking.

The aim of the present study was to characterize the effect of VRA in crop load management on fruit quality. The objectives were (1) to analyze the inter-year variability in LA and FBC of each individual tree considering their spatial position within a commercial orchard and (2) to generate the minimum thresholds of absorbed photons per fruit for each individual tree to achieve a desired mean fruit size and SSC.

## Materials and Methods

### Experimental Site and Trial Design

In 2018 and 2019, trials were carried out on trees of *Malus* x *domestica* Borkh. ‘Gala’ strain ‘Baigent’ (Brookfield^®^)/M.9 planted in 2006 in a commercial orchard in the fruit-growing region of Brandenburg, Germany (52.607 N, 13.818 E). The 2.3-m slender-spindle trained trees planted at a spacing of 3.2 × 1.0 m, with 3.2 m^2^ allotted orchard surface per tree (G_allotted_). Trees were drip-irrigated (<4 L tree^−1^ d^−1^), and managed according to the federal regulations of integrated production preventing any symptoms of nutrient- or water-deficit stress. Soil information were published earlier (Tsoulias et al., 2020b). Trees of five rows (199–200 trees row^−1^) of the orchard were labeled and analyzed. In the green bud stage, trees (2018: *n* = 100; 2019: *n* = 70) were randomly selected and the number of flower clusters per tree was counted. All trees were thinned chemically with ammonium thiosulphate (20% N; 15 kg ha^−1^) at full bloom (April 29, 2018 to April 24, 2019) and with 6-benzyl adenine (500 g ha^−1^) three weeks after full bloom. Subsequently, to generate variable numbers of fruit per tree, 60 trees of the selected samples were hand thinned to low (60 fruit tree^−1^), medium (100 fruit tree^−1^), and high (140 fruit tree^−1^) crop load each year. The average annual yield of the previous years was 50 t ha^−1^, which would equal to 106 fruit per tree on the 3,125 trees per hectare when targeting a fruit of 150 g FM at harvest.

At time intervals of 13–30 d during fruit development, starting 30 d after full bloom (DAFB) in both years until harvest, 30 randomly chosen apples from random trees were picked in the early afternoon and stored at 10 ± 2°C until the next morning when respiration rate, dry matter, and C content were measured for estimating the daily carbon requirements during fruit development.

At commercial harvest (September 3, 2018 to September 9, 2019), randomly selected apples (2018: *n* = 180; 2019: all fruit from nine trees, *n* = 1,240) were picked on one day and stored at 10 ± 2°C until the next morning for measuring fruit quality. Additionally, each apple of labeled trees (2018: *n* = 100; 2019: *n* = 70) was harvested and measured by using a commercial grading line.

During both seasons, the leaf CO_2_ gas exchange rate was recorded several times on the trees also sampled for fruit analysis. When the canopies were fully developed in July, the total LA per tree from all trees of the five rows (*n* = 996) was estimated from the three-dimensional (3D) point clouds recorded with a tractor-mounted LiDAR laser scanner. The estimations were based on a regression model of LiDAR points per tree (PPT) and the manually measured total LA of 16 trees with a LA meter (Tsoulias et al., 2021).

### Analyses of Fruit Growth, CO_2_ Gas Exchange, and Quality

Fruit diameter (D) and FM were measured by electronic caliper (Type 1108, INSIZE, Suzhou, China) and an electronic balance (CPA22480CE, Sartorius AG, Goettingen, Germany), respectively. The CO_2_ release rate providing the dark respiration rate (R_dT_) of 30 apples was measured by an IR CO_2_ gas analyzer (FYA600CO2, Ahlborn Mess- und Regelungstechnik GmbH, Holzkirchen, Germany) in a self-build closed system (Linke et al., 2010; Huyskens-Keil and Herppich, 2013). R_dT_ was measured at various temperatures (2018: 10 ± 2°C; 20 ± 2°C; 2019: 10 ± 2°C; 20 ± 2°C at 50 DAFB, 5 ± 2°C−25 ± 2°C in five°C-steps at 56, 103, and 138 DAFB) after 2 h of temperature acclimation between the measurements.

To quantify the daily amount of C respired per fruit, the dark respiration rates measured in the lab were utilized to generate a model of R_dT_ of DAFB and *T*_mean_. The rate of in field fruit respiration (R_d;field_) was estimated by using the model for the temperature measured in the field (*T*_mean)_, neglecting diurnal variations R_d;field_, which was used to calculate the daily respiratory C losses per fruit (^R^C_daily_, g d^−1^) with a factor 0.27 representing relative mass contribution of C in CO_2_ (Equation 1).

(1) CRdaily = Rd;field × FM × 24 × 0.27

Subsequently, the fruit was dried to constant mass (dry mass, DM) at 80°C. From DM and FM, the dry matter fraction (DM_rel_) was calculated as the ratio of FM to DM.

Dry matter samples were homogenized by using a mixer mill (MM400, Retsch Technology, Haan, Germany), and aliquots (10 mg) of the homogenized DM were analyzed for their relative C contents (C_rel_) with an element analyzer (Vario EL III, Elementar Analysensysteme GmbH, Hanau, Germany) at an operational temperature of 1,150°C. The absolute C content per fruit (C_fruit_, g) was calculated as

(2) $$\begin{array}{l} {\text{C}}_{\text{fruit}}\;=\text{ FM }\times \;{\text{DM}}_{\text{rel}}\;\times \;{\text{C}}_{\text{rel}} \end{array}$$

In commercial harvest, the SSC of individual fruit was analyzed with a digital refractometer (DR-301-95, Krüss, Hamburg, Germany) and fruit flesh firmness with a texture analyzer (TA.XT, Stable Micro Systems Ltd., Godalming, UK; 11.1 mm Magness-Taylor probe). Flesh firmness was obtained as the maximum force (N) at 10 mm penetration.

In addition, fruit harvested from the labeled trees (2018: *n* = 100; 2019: *n* = 70) were analyzed to capture fruit mass, color, yield per tree, and the number of fruit per tree with a commercial grader (GeoSort, Greefa, Tricht, The Netherlands).

### Leaf CO_2_ Gas Exchange

In both seasons (2018: 25, 58, 82, and 99 DAFB; 2019: 40, 47, 97, and 113 DAFB), light responses of steady-state leaf gas exchange were measured on three mature spur leaves from the bearing shoots of three randomly selected trees from each of the three crop load classes (*n* = 9 leaves per measurement date) with a portable gas exchange system (LI-6400 XT with the LI-6400-40 red/blue LED light source, LI-COR Inc., Lincoln, USA). At ambient leaf temperature (T_leaf_), relative humidity, and a constant CO_2_ mole fraction (400 μmol mol^−1^ in the reference gas), analyses were performed at photosynthetic photon flux rate (PPFR) of 2,000, 250, 100, 50, 20, and 0 μmol m^−2^ s^−1^ with the minimum waiting time of 100 s before each measurement. Maximum quantum yield (^max^α, mol mol^−1^) and the rate of light saturated CO_2_ gas exchange (^max^J_CO2_, μmol m^−2^ s^−1^) were analyzed (Matyssek and Herppich, 2017).

### Measurement of LA per Tree

Bud break was recorded on March 22, 2018 and on March 18, 2019. The canopy LA was assumed to be fully developed after 1,200 growing degree days after bud break (base temperature = 4°C; Doerflinger et al., 2015) on July 13, 2018, 80 DAFB and July 7, 2019, 84 DAFB. In the stage of fully developed canopy, all trees (*n* = 996) of the five labeled rows were scanned by using a mobile two-dimensional (2D) LiDAR laser scanner (LMS511 pro model, Sick, Düsseldorf, Germany) at a scanning frequency of 25 Hz and a vertical scanning angle of 270°. The LiDAR laser scanner was mounted on a tractor at 1.6 m height, together with an inertial measurement unit (MTi-G-710, XSENS, Enschede, The Netherlands) and an RTK-GNSS positioning system (AgGPS 542, Trimble, Sunnyvale, CA, USA) as described previously (Tsoulias et al., 2019). The sensor system was driven (0.13 m s^−1^) along both sides of the trees, acquiring the 3D point cloud of each individual tree for the five rows.

For tree segmentation, the position of each tree trunk was located from the bivariate density histograms of LiDAR points with an in-house developed (Tsoulias et al., 2019) Matlab script (Version 2018b, The Mathworks Inc., Natick, MA, USA). A vertical cylinder with a radius of 50 cm was projected based on the trunk position. The points within the cylinder boundaries were considered to belong to this tree and referred to as LiDAR PPT. Reference trees were defoliated after LiDAR scanning, and the area of each individual leaf was measured by using a LA meter (CI-203, CID Bio-Science, Camas, WA, USA). The regression model to convert PPT into total LA per tree (LA_LiDAR_, m^2^) from Tsoulias et al. (2021), which was established from the PPT of reference trees (*n* = 6 in 2018; *n* = 7 in 2019) and the manually measured total LA (Equations 3 and 4), was utilized to convert PPT of each tree into LA_LiDAR_.

(3) L2018ALiDAR(m2) =9.719 × 10−5 × PPT+1.84

(4) L2019ALiDAR(m2) =11.712 × 10-5 × PPT+0.75

### Modeling of Fruit FM and C-Requirement for Target Fruit Diameter

Seasonal changes of FM and C_fruit_ were interpolated over time (DAFB) by using a sigmoid-growth model. To derive the growth curves of apples by considering four harvest fruit diameters (65, 70, 75, and 80 mm), the growth equations based on the mean fruit FM and C_fruit_ were normalized with the measured mean FM and C_fruit_ at harvest. The growth curves of FM and C_fruit_ for target fruit diameters (D) were obtained (Equations 5 and 6) by multiplying the normalized growth functions with the target fruit diameter at harvest and a conversion regression equation from D to FM (Supplementary Figure 1).

(5) $$\begin{array}{l} \text{FM}\left(\text{g}\right)={\text{FM}}_{\text{norm}}\left(\text{DAFB}\right)\;\times \text{ FM}\left(\text{D}\right)\;\times \text{ D} \end{array}$$

(6) $$\begin{array}{l} {\text{C}}_{\text{fruit}}\left(\text{g}\right)={\text{C}}_{\text{norm}}\left(\text{DAFB}\right)\;\times \text{ FM}\left(\text{D}\right)\;\times \text{ D }\times \;{\text{DM}}_{\text{rel}}\;\times \;{\text{C}}_{\text{rel}} \end{array}$$

The first derivation of the resulting growth functions provided the absolute growth rates (AGR, g d^−1^) considering FM (AGR_FM_) and C_fruit_ (AGR_C_). The integral of AGR_C_ over time in DAFB provided the amount of C representing the fruit growth. The sum of AGR_C_ and ^R^C_daily_ denotes the daily C-requirement per fruit. The LA “demanded” (LA_demand_, cm^2^) to assimilate ∑*R*_*C*_*daily*__+ *AGR*_*C*_ was estimated (Equation 7; Penzel et al., 2020) for each tree sampled in the orchard and modeled for varying LA per tree (3.6, 5.5, and 7.7 m^2^ represented the 5th, 50th, and 95th percentile of the measured LA_LiDAR_, respectively). Daily fluctuations in LA_demand_ were smoothed by a Savitzky–Golay filter, using the R-Package “signal” (Ligges et al., 2015; sgolayfilt, filter order = 1, filter length = 9). P_daily_ (g m^−2^ d^−1^) reflects the C assimilated per unit soil area per day (Equation 8).

P_daily_ was calculated (Equation 8) as reported earlier (Lakso and Johnson, 1990; Penzel et al., 2020). P_daily_ was scaled up for the whole tree (P_tree_, g d^−1^) by multiplying it with G_allotted_, which was 3.2 m^2^ in equal planting distance of the orchard. C_part_ is a variable carbon-partitioning factor for the fraction of the assimilated carbohydrates partitioned to fruit; it was set to 0.8 when the foliage of trees was fully developed (Xia et al., 2009; Lakso, pers. communication). ∑^R^C_daily_ + AGR_C_ was generally reduced by 5% to roughly correct for fruit photosynthesis (Jones, 1981).

(7) $$\begin{array}{l} {\text{LA}}_{\text{demand}}\left(\text{c}{\text{m}}^{2}\right)=\frac{0.95\;\times \;\left(AGR_{C}\;+{\;}^{R}C_{daily}\right)}{\left(\frac{P_{daily}\;\times \;C_{part}}{LAI_{orchard}\;\times \;10,000}\right)} \end{array}$$

The daily integral of solar radiation (S, MJ m^−2^ d^−1^) was recorded by using a pyranometer (CMP 3, Kipp & Zonen, Delft, The Netherlands) in the spectral range of 300–2,800 nm. The day length (DL, s) was obtained by considering the daily hours with S > 0. The seasonal means of ^max^α and ^max^J_CO2_ were converted into energy units with the conversion factor of PPFR to S in direct sun light (0.4376; McCree, 1972). P_T_ (Equation 9) is a correction for the temperature dependence of ^max^J_CO2_, which was provided by Lakso (pers. communication), utilizing the mean temperature of the daily hours when S > 0 (T_mean, day_). The fraction of light intercepted by the canopy (LI) was calculated (Equation 10) by considering the canopy light extinction coefficient (k) and the fraction of total radiation incidence on the canopy (LI_max_), which were set to 0.5 (Poblete-Echeverría et al., 2015) and 0.7 (Doerflinger et al., 2015), respectively. The individual LA index of trees in the orchard (LAI_orchard_) was calculated by dividing LA_LiDAR_ with G_allotted_ (Equation 11).

(8) $$\begin{array}{l} {\text{P}}_{\text{daily}}\left(\text{g}{\text{d}}^{-1}\right)=\frac{{max}_{\alpha }\;\times \;S\;\times \;DL\;\times {\;}^{max}JCO2\;\times \;PT\;\times \;LI}{{max}_{\alpha }\;\times \;k\;\times \;S\;+\;DL\;\times {\;}^{max}JCO2\;\times \;P_{T}\;}\\ \;\times \;0.27 \end{array}$$

(9) $$\begin{array}{l} {\text{P}}_{\text{T}}\left[0-1\right]=0.535+0.0384\times T_{\text{mean},\text{day}}-0.0004126\\ \;\times {T_{\text{mean},\text{day}}}^{\text{2}}-0.00001576\times T_{\text{mean},\text{day}}3 \end{array}$$

(10) $$\begin{array}{l} \text{LI}\left[0-1\right]={\text{LI}}_{\text{max}}\;\times \;\left(1\;-\;{\text{e}}^{\left(-\text{k }\times \;\frac{LAI_{orchard}}{LI_{max}}\right)}\right) \end{array}$$

(11) $$\begin{array}{l} {\text{LAI}}_{\text{orchard}}=\frac{LA_{LiDAR}}{G_{allotted}} \end{array}$$

### Modeled FBC

The FBC (Equation 12) of 996 trees was calculated by considering the LA_demand_ for the four target fruit diameters, and the actual LA was analyzed by using LiDAR (LA_LiDAR_). The ratio was built for the relevant time of 15 d before and after the climax of fruit growth.

(12) $$\begin{array}{l} \text{FBC}\left(\text{fruit tre}{\text{e}}^{-1}\right)=\frac{LA_{LiDAR}}{LA_{demand}} \end{array}$$

### Total Absorbed Photosynthetic Energy

The total absorbed photosynthetic energy (TAPE, MJ; Equation 13) of each tree was considered for the period of fully developed canopy (FDC) LA until harvest. TAPE was divided by the number of fruit per tree for obtaining the TAPE per fruit (MJ fruit^−1^).

(13) $$\begin{array}{l} \text{TAPE}\left(\text{MJ}\right)=\text{LI}\times \sum_{\text{FDC}}^{\text{Harvest}}\;S\;\times \;0.5\;\times \;{\text{G}}_{\text{allotted}} \end{array}$$

The incident photosynthetic active photon flux rate was estimated by multiplying LI for each individual tree (Equation 10) with the integral of solar radiation, S, with the assumed fraction of PAR on solar radiation (0.5; Szeicz, 1970), and with G_allotted_ (3.2 m^2^ in the present orchard).

### Statistical Analyses

Descriptive analysis capturing regression analysis and ANOVA were carried out in R (Version 3.4.1, R Core Team, 2018). CI of 95% was used. The value of *p* < 0.05 was considered as significant. The FBC of each individual tree was visualized by Getis–Ord's analysis at confidence levels ≥90% (c.f. Peeters et al., 2015) calculated in ArcGIS (v.10.2.1, ESRI, Redlands, CA, USA). Hot spot and cold spot analysis (Peeters et al., 2015) indicate trees or clusters of trees having either a very high (hot spot) or a very low (cold spot) *Z* score, either < -1.96 or >1.96, reflecting low and high FBC, respectively.

## Results

### Fruit Development and Its C-Requirement

**Figure 1:**
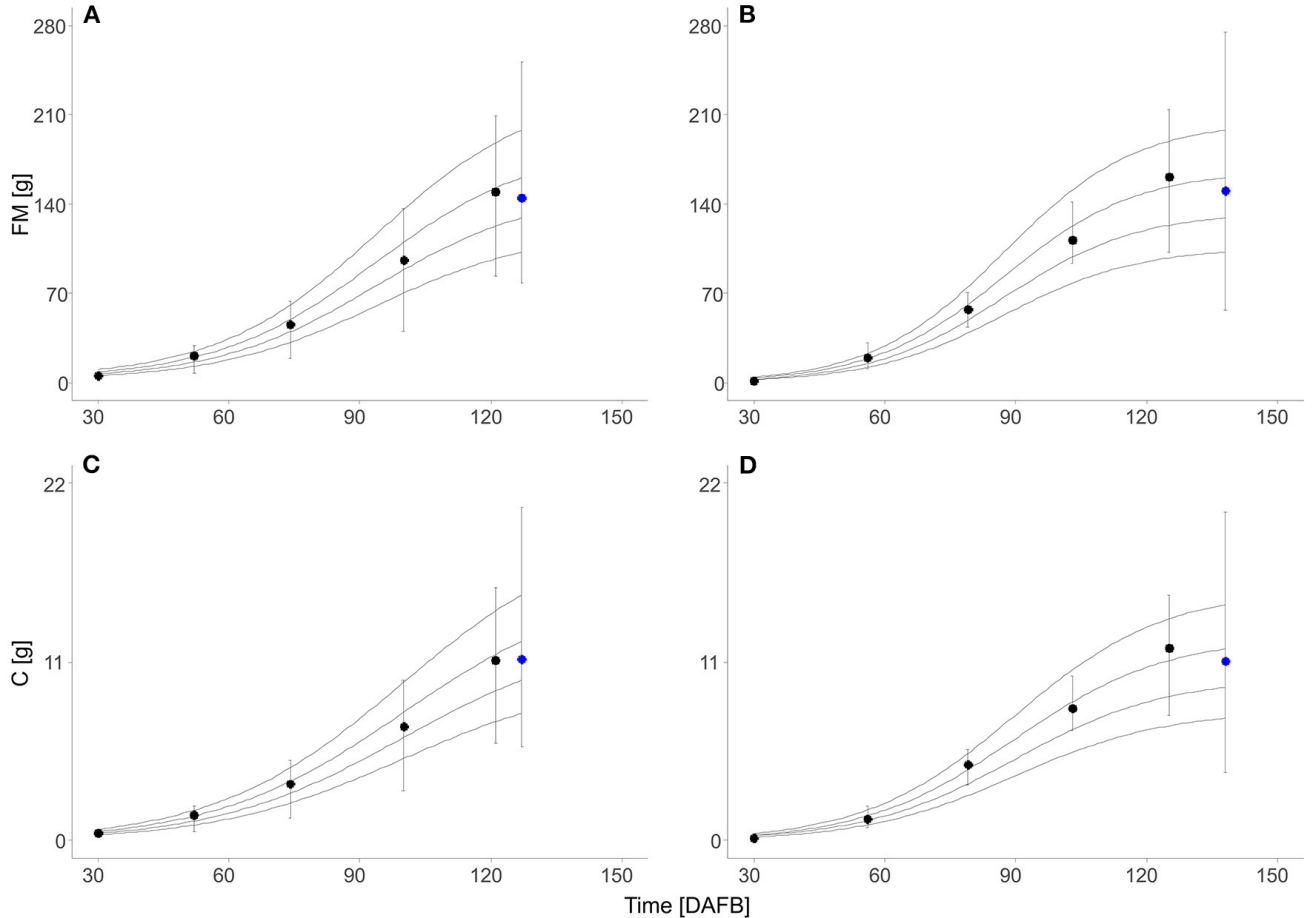
Fresh mass (FM) **(A,B)** and absolute C content (C_fruit_) **(C,D)** of ‘Gala’/M.9 apples during the season (black circle, *n* = 30) and at harvest (blue circle, 2018: *n* = 180; 2019: *n* = 1,240) in days after full bloom (DAFB) in 2018 **(A,C)** and 2019 **(B,D)**. Symbols represent the measured means, error bars show the *SD*, and solid lines show the sigmoid-growth functions simulated for the fruit with 65, 70, 75, and 80 mm diameter at harvest (from the bottom to top).

Fruit development from full bloom to harvest in the beginning of a climacteric peak lasted 11 d longer in 2019 (127 d) than in 2018. Nevertheless, the mean fruit FM at harvest was similar in both years (2018: 145 g; 2019: 150 g). Sigmoid-growth functions were applied to interpolate the measured values of FM and C_fruit_ and model the increase of FM and C_fruit_ during fruit development (Supplementary Equations 1, 2). From the normalized equations, sigmoid-growth curves were calculated by considering the four target fruit diameters. The simulated growth curves showed a horizontal shift explaining the difference of fruit FM and C content multiplicative distributed over the season (Figure 1).

Some data necessary for modeling the FBC, but not relevant to point out the new findings on the effect of measured LA on FBC, were presented in the Supplementary Material: The fraction of DM on fruit FM with a mean of 0.15 in both seasons (Supplementary Figure 2). The carbon content in the fruit DM, C_rel_, decreased from 0.51 at 30 DAFB to 0.48 at harvest (Supplementary Equation 3 and Supplementary Figure 2). The maximum values of the absolute fruit growth rate considering C content, AGR_C_, were found at 101 DAFB in 2018 and 92 DAFB in 2019 (Supplementary Figure 3). The fruit dark respiration rate decreased during fruit development and increased with temperature (Supplementary Figure 4). At the last measurement date of harvest, R_dT_ was slightly enhanced in comparison to the two measurement dates before, indicating the onset of a climacteric rise in fruit respiration. Temperature-corrected ^R^C_daily_ increased during fruit development due to enhanced temperature in the orchard. However, the respiration-related fraction, ^R^C_daily_, showed a high daily fluctuation (Supplementary Figure 5) ranging from 5 to 15% in 2018 and from 3 to 28% in 2019. Considering fruit of the same target diameter, the modeled total amount of respiratory C loss from 50 DAFB till harvest was in a similar range for 2018 (0.57–1.11 g) and 2019 (0.65–1.26 g). This fruit respiration accounted for 7% (2018) and 10% (2019) of the carbon requirement of fruit in the considered period.

**Figure 2:**
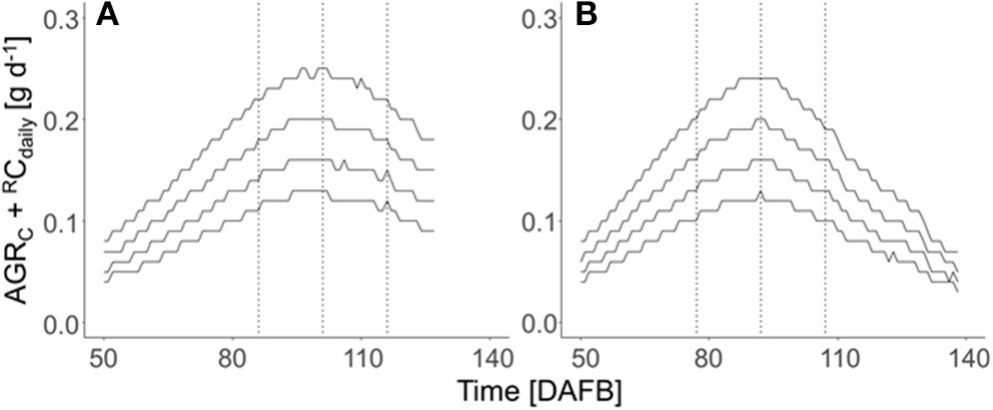
Seasonal course of the sum of the C-based daily fruit growth rate (AGR_C_, g d^−1^) and daily respired C per fruit (^R^C_daily_, g d^−1^) of ‘Gala’/M.9 apples with target diameters of 65, 70, 75, and 80 mm (from the bottom to top) during the DAFB in 2018 **(A)** and 2019 **(B)** in DAFB. The dotted vertical lines represent the period ±15 d of the fruit's highest daily C-requirement (2018: 86–116 DAFB; 2019: 77–107 DAFB).

The total carbon requirement of apples (Figure 2) was calculated by the sum of respiratory C loss and fruit growth, which considers four target diameters in the period after cell division till harvest. A horizontal shift of the sum of AGR_C_ + ^R^C_daily_ appeared for the four target fruit diameters when assuming a similar fruit growth over the season. The total fruit carbon requirement was slightly higher in 2018 than in 2019 (Table 1). The seasonal maximum in the carbon requirements per fruit appeared at 92 and 101 DAFB in 2018 and 2019, respectively (Figure 2). The period ±15 d from the seasonal maximum in the carbon requirements per fruit in both years (Figure 2) was considered for estimating the FBC of the trees.

**Table 1:** Total fruit C demand calculated from the sum of absolute C-based growth rates (AGR_C_) and respiratory C loss (^R^C_daily_) of ‘Gala’/M.9 apples of the four targeted fruit diameters for the period of 50 d after full bloom (DAFB) till harvest in 2018 and 2019.

	$\overset{Harvest}{\underset{50\;DAFB}{\sum }}\left(AGR_{C}{+}^{R}C_{daily}\right)\;\left(g\right)$	
**Target fruit diameter (mm)**	**2018**	**2019**
65	7.6	7.5
70	9.5	9.4
75	11.9	11.8
80	14.7	14.5

### LA and Canopy Carbon Assimilation

**Figure 3:**
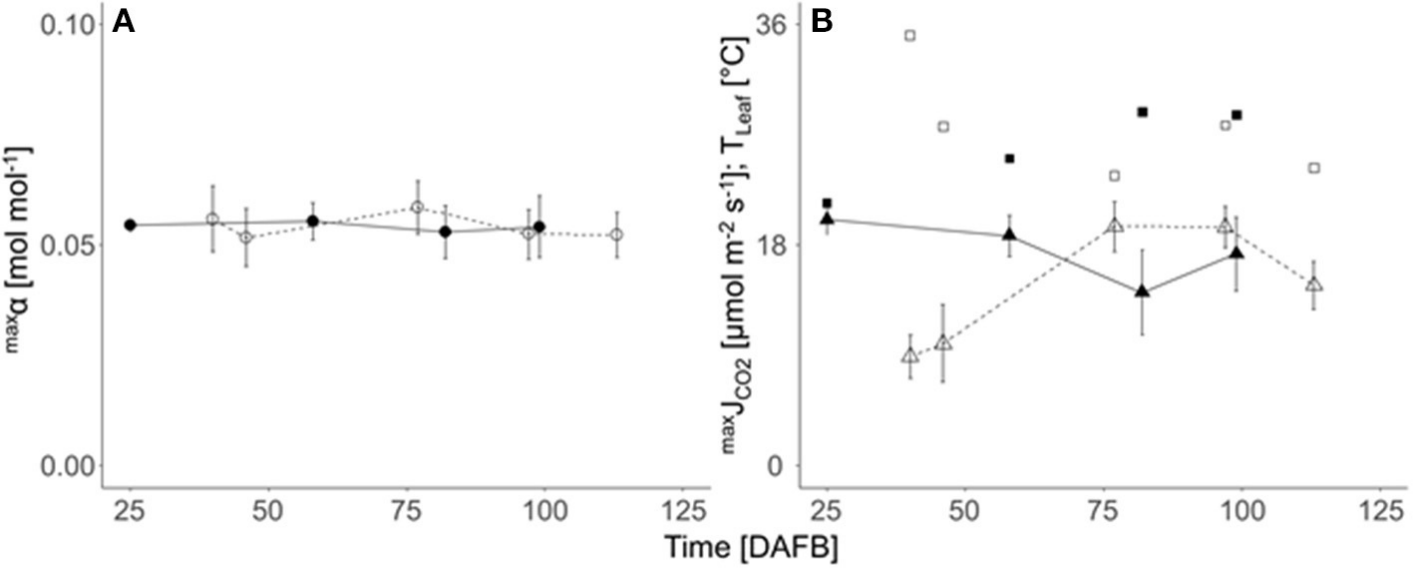
Seasonal course (DAFB) of means (± *SD*; *n* = 9) of **(A)** the maximum quantum efficiency of photosynthesis (^max^α, circles), **(B)** light saturated maximum CO_2_ gas exchange rate (^max^J_CO2_, triangles) and leaf temperature (T_leaf_, squares) of fully developed ‘Gala’/M.9 apple spur leaves in 2018 (closed symbols, solid lines) and 2019 (open symbols, dashed line).

The maximum quantum efficiency of leaf photosynthesis (^max^α) was not affected by either crop load, leaf temperature, season or the actual leaf-to-air partial pressure deficit for water vapor (Δw) in both years (not shown) and varied marginally during the season as well as between both seasons (Figure 3A). Therefore, the overall mean of ^max^α 0.054 mol mol^−1^ was considered in all calculations (Equation 8). The seasonal variation of ^max^J_CO2_ (Figure 3B) was slightly higher than that of ^max^α, due to the stomatal effects mainly caused by pronounced seasonal changes in Δw (data not shown). In 2019, at 40 and 46 DAFB, the ratio between leaf internal (c_i_) and ambient CO_2_ concentrations (c_a_), pointing to the degree of stomatal limitation of ^max^J_CO2_, was lower (0.06–0.22) than the ratio of the measured 77 and 97 DAFB (0.62–0.85). A regression of ^max^J_CO2_ against stomatal conductance showed non-stomatal limited ^max^J_CO2_ at 19.8 μmol m^−2^ s^−1^, which was applied in both years.

**Figure 4:**
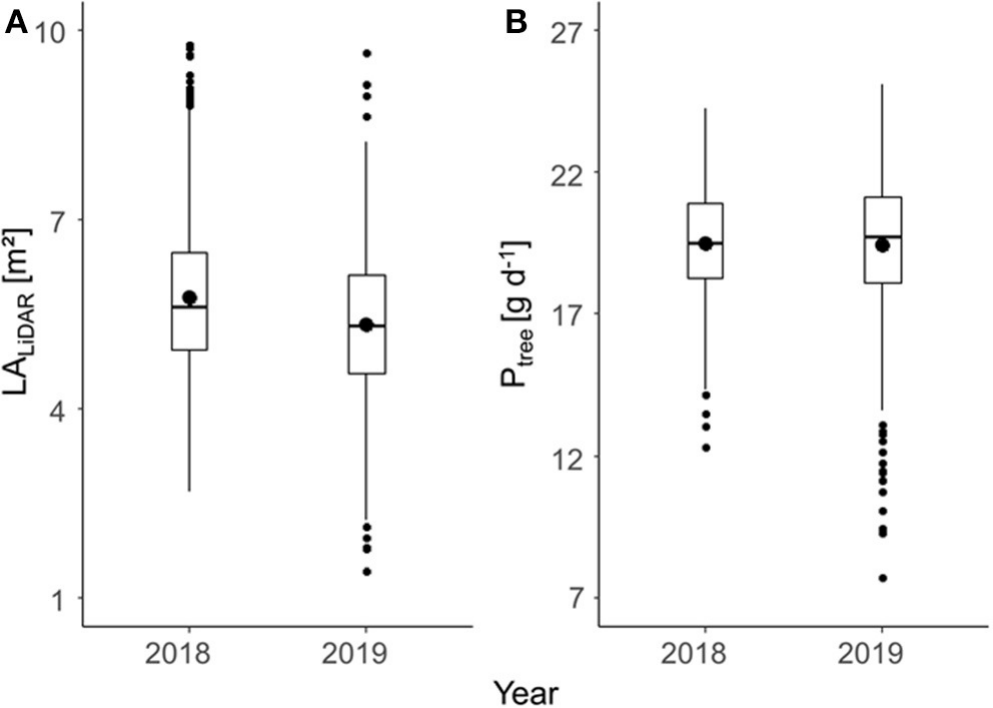
Total leaf area (LA) per tree estimated with light detection and ranging (LiDAR) (LALiDAR) of a fully developed canopy **(A)** and mean daily assimilated C per tree (Ptree) in the period of maximum daily fruit carbon requirement **(B)** of ‘Gala’/M.9 apple trees in two consecutive years. Lower and upper hinges of boxplots correspond to the first and third quartile, the dash inside the box to the median, and the dot to the mean value.

The mean LiDAR-estimated total LA per tree (LA_LiDAR_) was slightly higher in 2018 (5.8 m^2^) than that in 2019 (5.3 m^2^) (Figure 4A), which corresponds to the slightly enhanced mean daily assimilated C in 2018. The estimated fraction of incident light intercepted by the canopy (LI) estimated in Equation (10) ranged between 0.3 and 0.6 in 2018, and 0.2 and 0.6 in 2019, with a mean of 0.5 for both years. The temporal mean of assimilated C (g) was analyzed for each tree to point out the impact of LA on carbon gain without considering the fruit. P_tree_ was calculated for each tree in mean conditions (2018: DL = 14 h, S = 17.5 MJ m^2^ d^−1^, *T*_mean;day_ = 25.7°C; 2019: DL = 15 h, S = 17 MJ m^2^ d^−1^, *T*_mean;day_ = 21°C) for the period of the maximum fruit carbon requirement ±15 d (Figure 4B). The data on daily carbon gain per tree reflect the varying LA_LiDAR_ of each individual tree (Figure 4).

**Figure 5:**
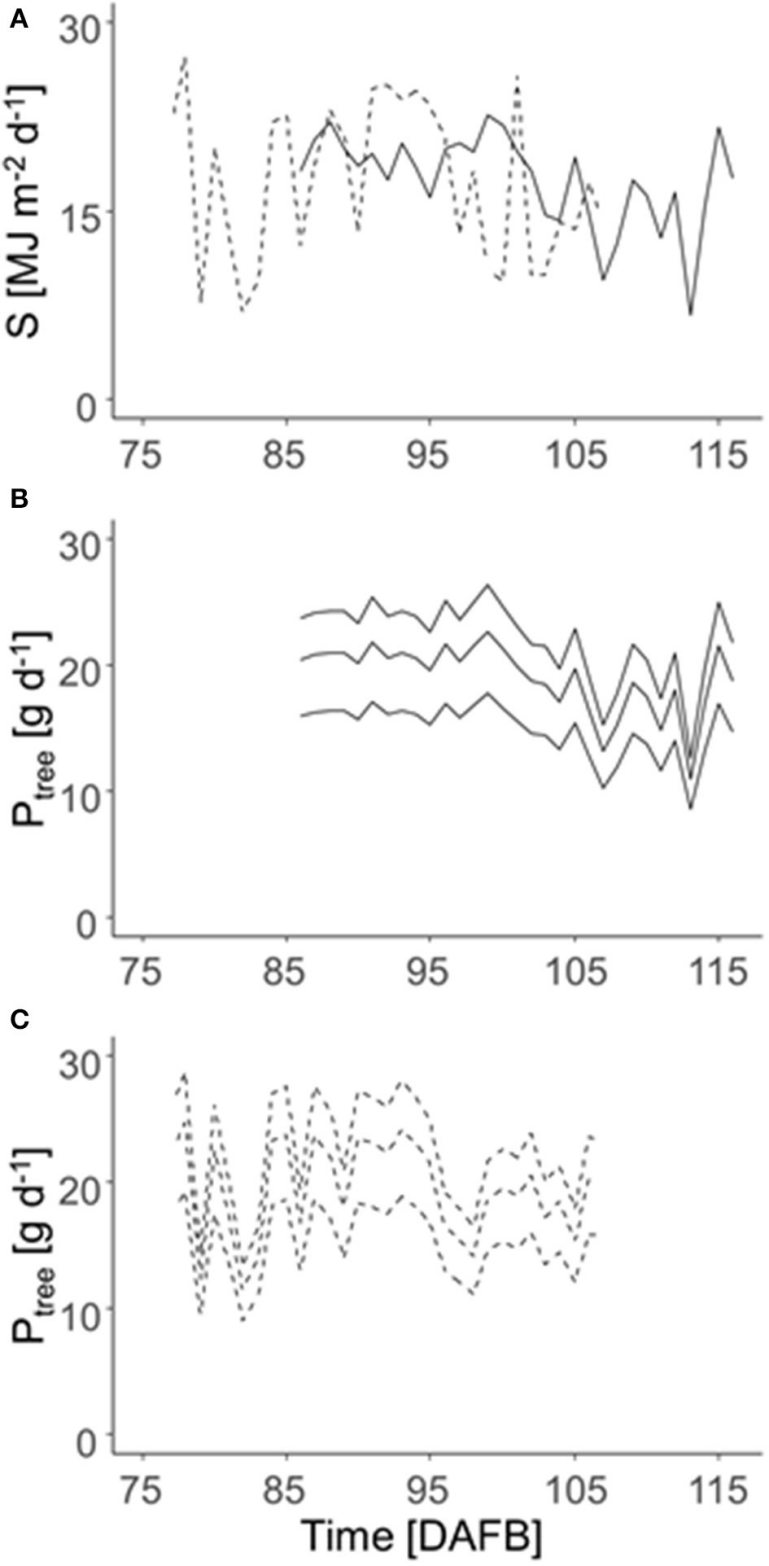
Daily integrated solar radiation (S) in 2018 (solid lines) and 2019 (dashed lines) **(A)** and daily C gain per tree (P_tree_) of ‘Gala’/M9 during the time of maximum daily fruit C-requirement (2018: 86–116 DAFB; 2019: 77–107 DAFB) (**B**: 2018; **C**: 2019). P_tree_ was calculated for the trees of 3.6, 5.5, and 7.7 m^2^ total LA (lines from the bottom to top), which represents the 5, 50, and 95th percentile of LA_LiDAR_ of all 996 trees measured during both years.

The daily integral of solar radiation (S) was highly fluctuating in both years (Figure 5A). Consequently, the daily carbon gain of the trees (P_tree_) fluctuated pronouncedly during the relevant period of maximum carbon requirement by the fruit in both years (Figures 5B,C) with maximum values of 24 (2018) and 25 g d^−1^ (2019) considering the overall mean LA of 5.5 m^2^. Figure 5 points out the impact of low, mean, and high LA on the daily P_tree_. However, this analysis ignores the shading effects within the canopy.

### LA Demand and FBC Considering Target Fruit Diameters

**Figure 6:**
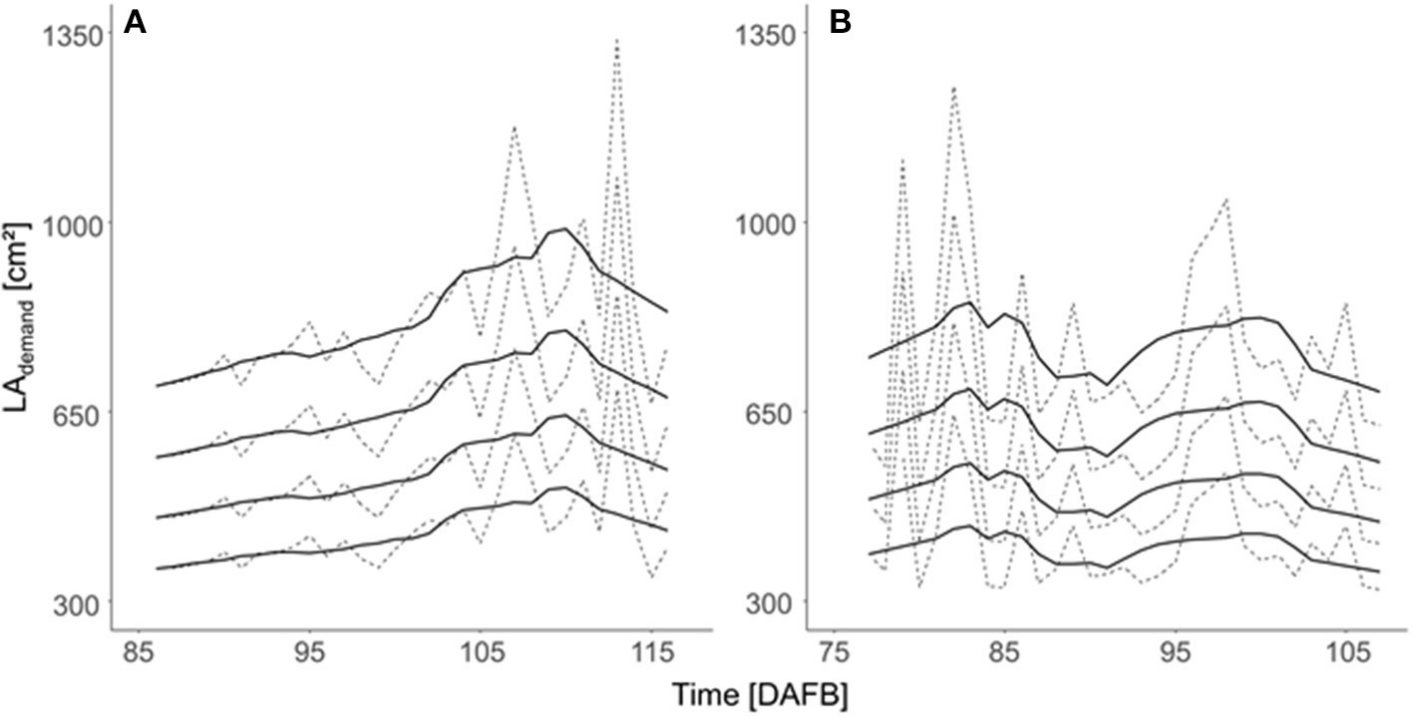
Daily LA demand [LA_demand_ (cm^2^)] per fruit estimated for the four target fruit diameters (lines from bottom to top: 65, 70, 75, and 80 mm) of ‘Gala’/M.9 trees considering the orchard's mean total LA of 5.5 m^2^ during the period 15 d before and after maximum daily fruit C-requirement in **(A)** 2018 and **(B)** 2019. Dashed lines represent the estimated daily values and solid lines are the values that are smoothed by using a Savitzky–Golay filter.

The LA demand per fruit (LA_demand_) varied during fruit development according to ^R^C_daily_ and P_daily_, which are affected by temperature and solar radiation (Figure 6). Assuming a uniform LA_demand_ for all trees in the orchard and one target fruit diameter, the daily LA_demand_ during the period of maximum fruit C-requirement was slightly higher in 2018 than in 2019.

**Figure 7:**
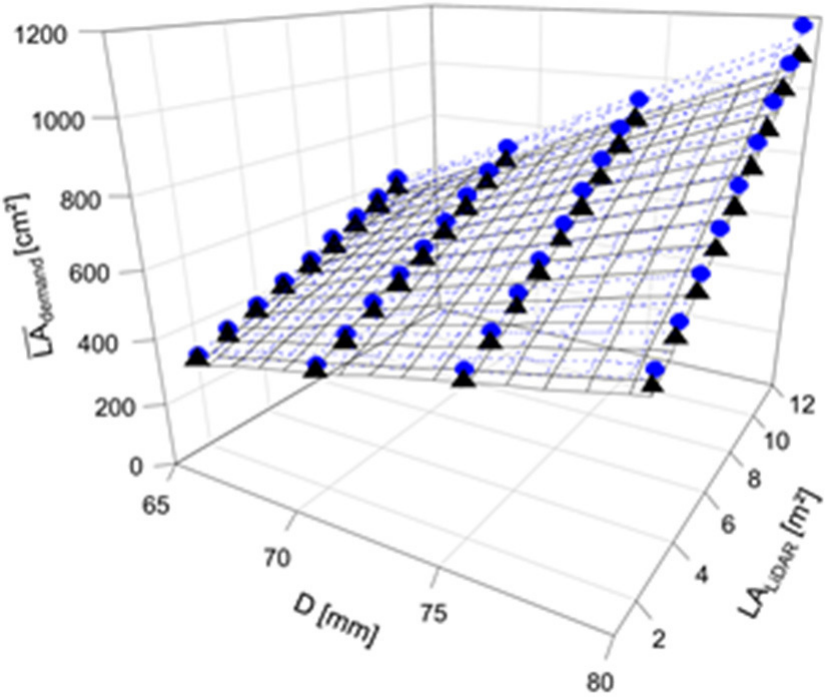
Mean LA demand per fruit (^2018^LA_demand_, ^2019^LA_demand_, cm^2^) considering the four target fruit diameters (D) of ‘Gala’/M.9 apple in 2018 (gray circle, dotted line) and 2019 (black triangle, solid line) for the trees with different total LAs (LA_LiDAR_, m^2^) in the period of 15 d before and after the highest daily C-requirement per fruit (2018: 86–116 DAFB; 2019: 77–107 DAFB).

Regression analyses were carried out to quantify the relationship between the mean LA_demand_ and the actual range of measured LA using LiDAR. The regression models provide the LA_demand_ necessary for the target fruit diameter in 2018 and 2019 (Table 2). LA_demand_ increased with the target fruit diameter (Figures 6, 7). Additionally, LA_demand_ was enhanced with increased actual LA_LiDAR_ (Figure 7). It can be assumed that a hyperbolic response of light interception to LA_LiDAR_ and the associated canopy density (Equation 10) caused this non-linearity.

**Table 2:** Regression equations of the relationship between the mean LA_demand_ and the light detection and ranging (LiDAR)-measured leaf area (LA_LiDAR_) for the estimation of the LA necessary to yield fruit of the target diameter (D) in 2018 and 2019.

**Year**	**Regression equation**	**Equation**
2018	LA_demand_ = −714.6813 + 14.4682 × D – 113.006 × LA_LiDAR_ + 2.2882 × D × LA_LiDAR_	14
2019	LA_demand_ = −667.0759 + 13.5078 × D – 105.5594 × LA_LiDAR_ + 2.1374 × D × LA_LiDAR_	15

The mean LA of each individual leaf, capturing the data from both years, was 21 cm^2^. Consequently, the number of leaves necessary per fruit would range from 12 to 57 leaves per fruit.

**Figure 8:**
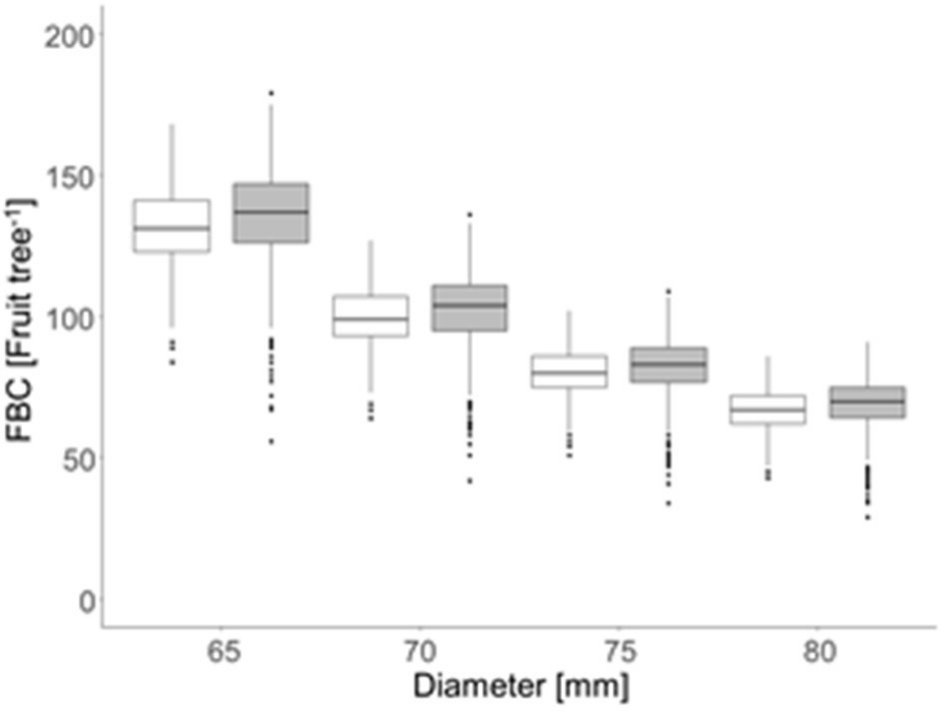
Mean fruit-bearing capacity [FBC (fruit tree^−1^)] considering the four target fruit diameters (D) of the ‘Gala’/M.9 apple in 2018 (open box plots) and 2019 (gray box plots) for 996 trees per year in the period of 15 d before and after the highest daily fruit C-requirement (2018: 86–116 DAFB; 2019: 77–107 DAFB).

The FBC, calculated for each tree by the ratio of LA_LiDAR_ and LA_demand_ conferring all four target fruit diameters, ranged from 43 to 168 apples per tree (2018) and 28 to 179 apples per tree (2019) (Figure 8). A maximum difference of 11 apples in FBC between both years for the trees with the same LA_LiDAR_ and fruit diameter was obtained.

**Figure 9:**
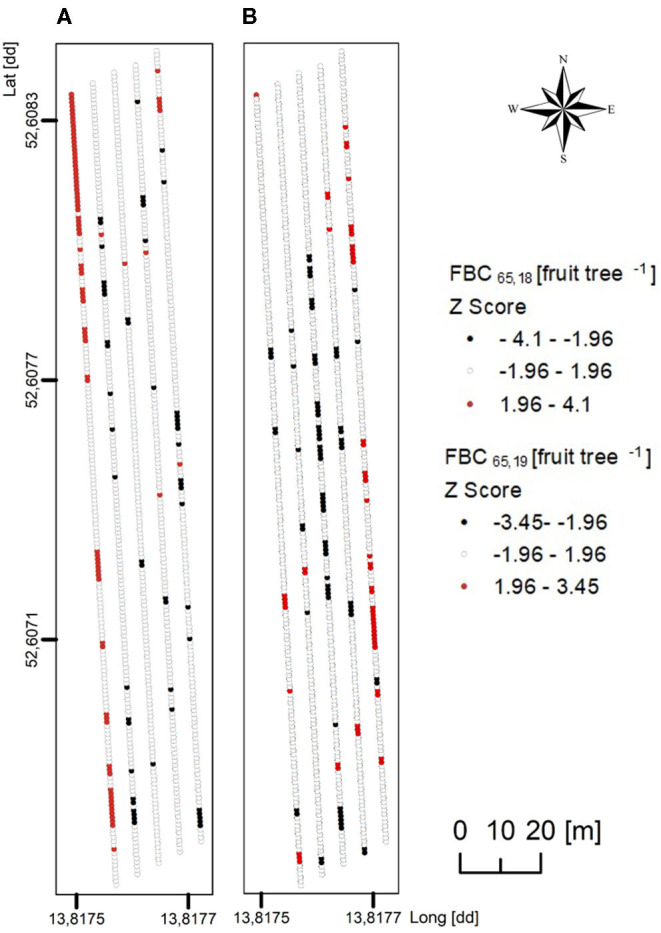
Maps of *z*-scores in Getis–Ord's analysis applied to FBC for the mean fruit diameter of 65 mm (FBC_65_), considering 996 trees of ‘Gala’/M.9 per year in **(A)** 2018 and **(B)** 2019. Red indicates significant spatial clusters of high values (a hot spot), black indicates significant spatial clusters of low values (a cold spot), and white indicates random distribution with no spatial clustering.

In the present orchard, the number of flower clusters per tree was highly variable with 50–220 in 2018 and 73–296 in 2019, sufficient for each tree to meet the FBC when assuming that a tree can generate one to two fruit per cluster at harvest. Trees (*n* = 996) were classified according to their FBC with *D* = 65 mm to locate the trees having a FBC_65_ below (cold spots) or above (hot spots) the majority of trees. The values of *Z* between −1.96 and 1.96 represented the majority of the trees having a mean FBC_65_ of 130 and 135 fruit tree^−1^ in 2018 and 2019, respectively. Cold spots showed a mean FBC_65_ of 110 and 106 fruit tree^−1^ whereas the trees representing hot spots had a mean FBC_65_ of 156 and 155 fruit tree^−1^ in 2018 and 2019, respectively (Figure 9). Despite the findings of high variability of LA and FBC in the orchard, the mean values of low, mean, and high crop load were similar. However, cold and hot spots were found in different locations by comparing both years. To conclude, the LA of a certain year cannot be used for predicting hot and cold spots of the following year.

The modeled FBC was validated by using measurements in a commercial grader at harvest, considering each individual tree (2018: *n* = 100; 2019: *n* = 70). The expected fruit diameter from the modeled FBC was compared to the measured values of fruit diameter in the grader: in both years, the actual number of fruit per tree having *D* > 65 mm and the calculated FBC, considering the actual average fruit diameter and LA_LiDAR_ per tree were similar as shown by their ratio (Table 3). The high *SD*, however, pointed out a high percentage of trees with crop load above or below the FBC.

**Table 3:** The ratio between the actual number of fruit per tree with diameter (D) >65 mm considering all fruit measured when harvesting whole trees and modeling fruit-bearing capacity (FBC) for the actual average fruit diameter at harvest of ‘Gala’ trees in 2 years.

**Year**	**Number of trees**	**Number of fruit with D > 65 mm per tree/FBC (mean ±*SD*)**
2018	100	0.97 ± 0.39
2019	75	1.05 ± 0.29

### Fruit Quality

**Figure 10:**
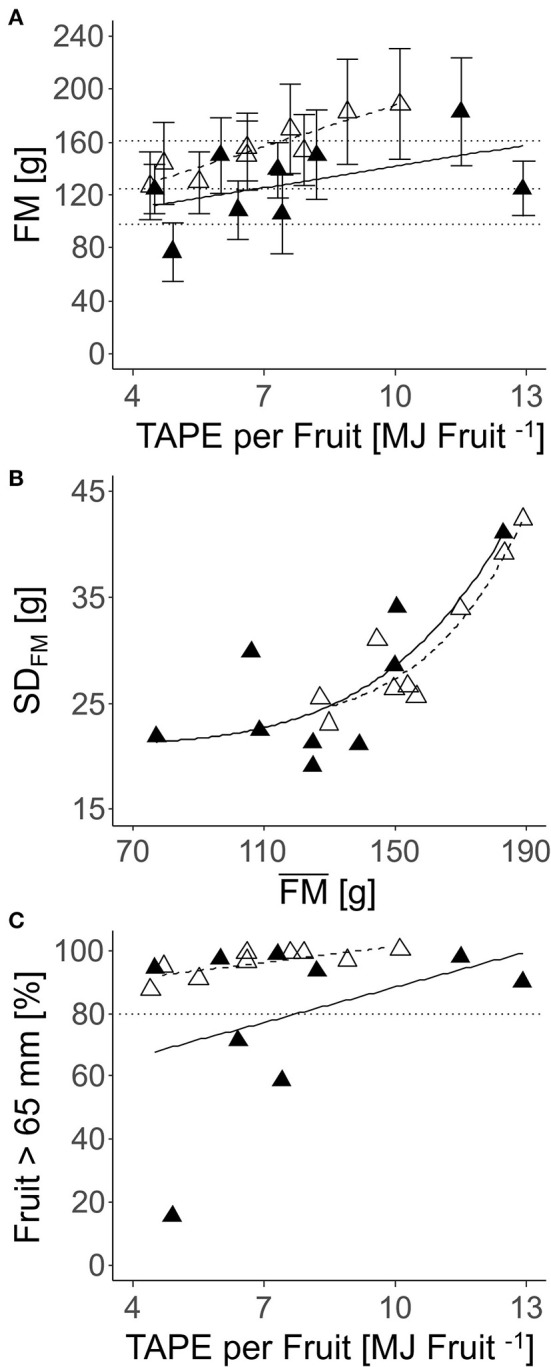
Relationships between FM (mean ± *SD*) per tree (*n* = 9) harvested completely and the number of fruit per TAPE per fruit (TAPE^*^ fruit^−1^) **(A)**; *SD* of FM and mean FM **(B)**; percentage of a fruit with *D* > 65 mm and TAPE fruit^−1^ **(C)** in: ‘Gala’ apples in 2018 [closed triangle, solid line; **(A)** *R*^2^ = 0.16; **(B)** *R*^2^ = 0.38; **(C)** not significant] and 2019 [open triangle, dashed line; **(A)** *R*^2^ = 0.23; **(B)** *R*^2^ = 0.77; **(C)** *R*^2^ = 0.89] (2018: 80–127 DAFB; 2019: 84–138 DAFB).

The effect of TAPE considering LA_LiDAR_ of each individual tree per fruit (Equation 13) on fruit quality was analyzed in the laboratory. FM and diameter were enhanced with increasing TAPE per fruit (Figure 10A). The FM of individual fruit showed high *SD*, which increased with average FM (Figure 10B). A high percentage of apples with *D* > 65 mm was found in all nine trees analyzed completely in the laboratory (Figure 10C).

**Figure 11:**
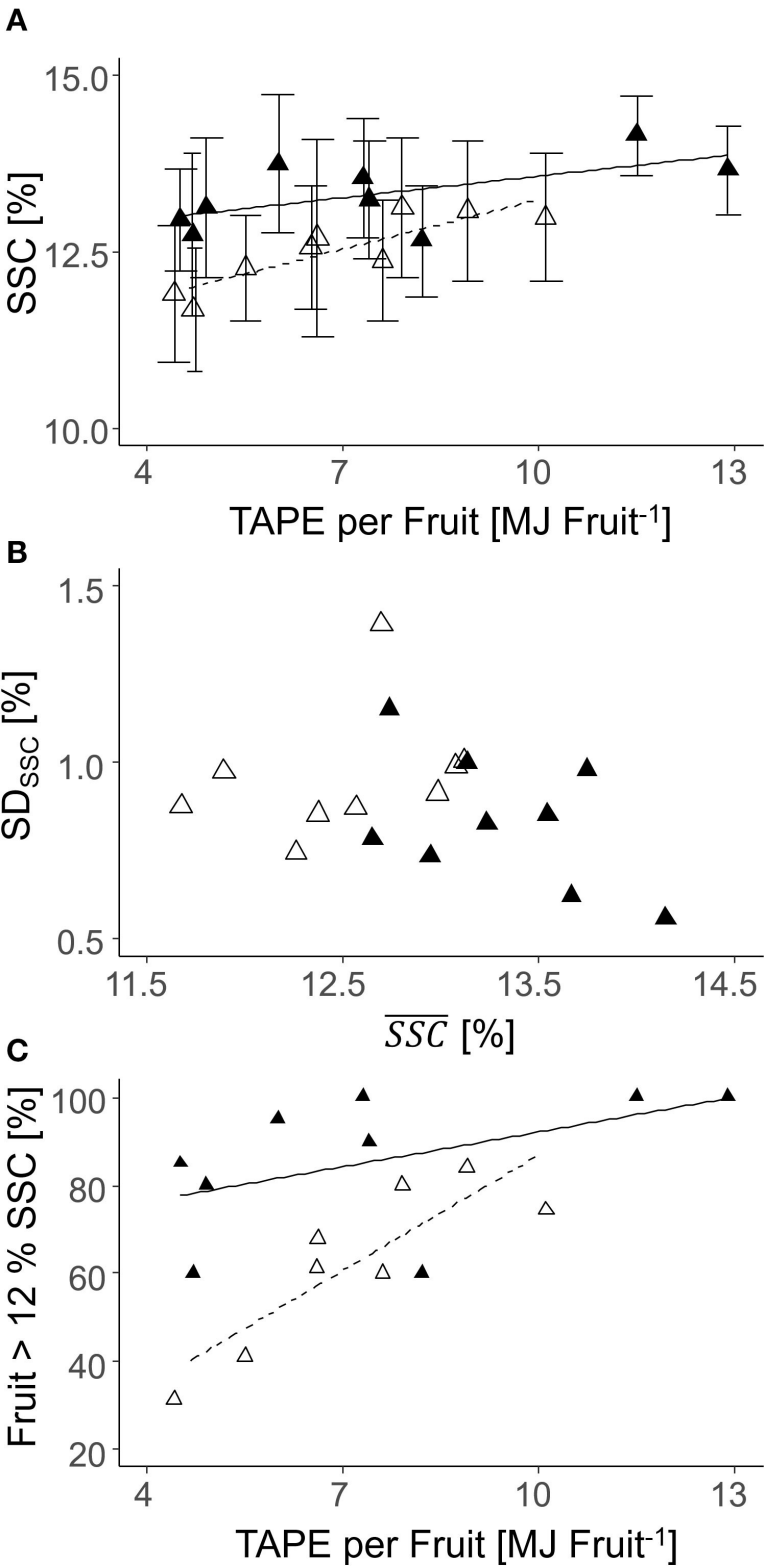
Relationships between the soluble solids content [SSC (%)] and the total absorbed photosynthetic energy (TAPE)^*^ per fruit **(A)**; *SD* and mean SSC **(B)**; the percentage of fruit with SSC > 12% per tree according to TAPE per fruit **(C)** of ‘Gala’ fruit from each individual tree (*n* = 9) in 2018 [closed triangle, solid line; **(A)** *R*^2^ = 0.16; **(C)** ns] and 2019 [open triangle, dashed line; **(A)** *R*^2^ = 0.23; **(C)** *R*^2^ = 0.73] (2018: 80–127 DAFB; 2019: 84–138 DAFB).

Fruit flesh firmness at harvest was 67 ± 9 N in 2018 and 86 ± 9 N in 2019, with a range between maximum and minimum values of 76 N (2018) and 86 N (2019) (Supplementary Table 2). TAPE per fruit had no effect on firmness in both years. In contrast, TAPE per fruit affected SSC but to a different extent comparing both years. The SSC was generally lower in 2019 than in 2018 (Figure 11). The SD of SSC was not related to the mean SSC at harvest (Figure 11B). Enhanced TAPE per fruit caused an increased percentage of fruit having SSC ≥ 12% from 30 to 80% in 2019 while the effect was less pronounced in 2018 (Figure 11C).

**Figure 12:**
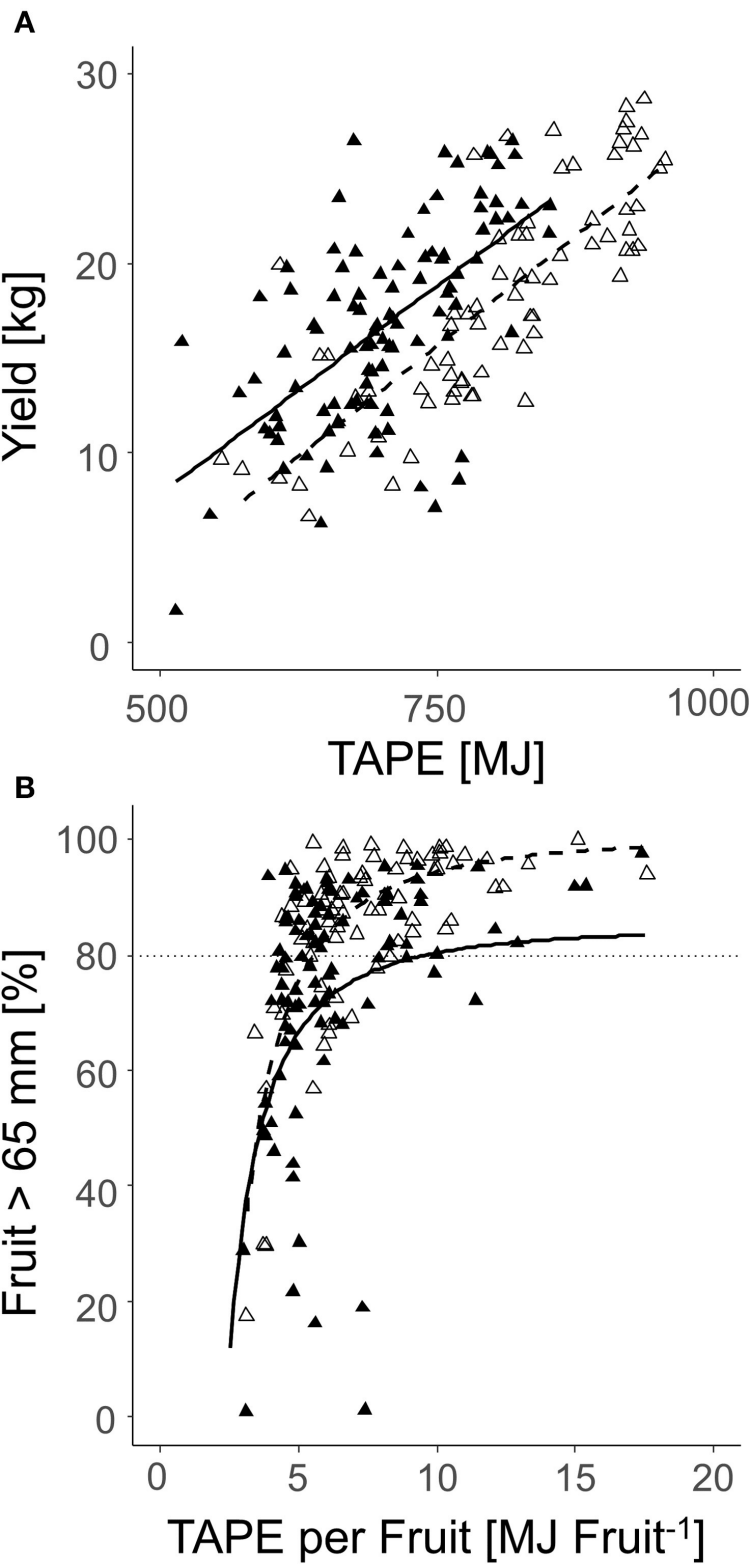
Relationship between the total yield per tree and TAPE^*^ (MJ) **(A)**; the percentage of the marketable fruit with D > 65 mm and TAPE per fruit (MJ fruit^−1^) **(B)** of ‘Gala’ in 2018 [closed triangle, solid line, **(A)** *R*^2^ = 0.40; **(B)** *R*^2^ = 0.25] and 2019 [open triangle, dashed line, **(A)** *R*^2^ = 0.63; **(B)** *R*^2^ = 0.58] (2018: 80–127 DAFB; 2019: 84–138 DAFB).

When all fruit per tree (2018: *n* = 100; 2019: *n* = 70) were analyzed on the sorting line, a correlation of total yield per tree and TAPE based on the LA_LiDAR_ was found. The *R*^2^ was enhanced in 2018 compared to 2019 (Figure 12A). Additionally, the percentage of fruit with D > 65 mm was correlated with TAPE per fruit (Figure 12B). In enhanced TAPE per fruit, more than 60% of the apples had a marketable fruit *D* > 65 mm. The slope of the curve indicated over 80% (2018) and 90% (2019) of the marketable fruit at 7.5 and 5.9 MJ fruit^−1^, respectively (Figure 12B).

High quality, considering the blush color, was defined as the fruit showing ≥60% red blush of the entire fruit surface measured with a commercial grader (Supplementary Table 2). In 86% of the trees, high-quality blush color occurred in 80% of the entirely harvested fruit. In 95% of the trees, at least 60% of the fruit showed a high-quality red blush. However, no effect of TAPE per fruit was found on blush color of the red ‘Gala’ strain in both years.

## Discussion

### Variability of FBC

This study aimed to model the FBC of each individual tree in a commercial orchard for two consecutive years. A considerable range of LA was found in the present study (Figure 4A). The LA differences correspond to the associated mid-season range of photosynthetic performance (Figures 4B, 5B) and, hence, the growth capacity of each individual tree to produce fruit. The FBC for the desired mean fruit diameter varies between 65 and 80 mm (Figure 8). The FBC was calculated by considering the period of seasonal maximum in fruit growth and the resulting maximum daily fruit C-requirement. In this period, the LA of the canopy is already fully developed.

The measured input data of the FBC model (fruit growth rate, fruit and leaf CO_2_ gas exchange rates) are in close agreement with the ranges reported in the previous studies on apple (Yuri et al., 2011; Baïram et al., 2019; Penzel et al., 2020). Enabling fruit to meet their maximum growth potential, which frequently refers to sink limited fruit growth (Reyes et al., 2016), is commercially always avoided. For maximum fruit growth rates, low crop loads are required, which lead to low yield and possible physiological disorders of fruit (Ferguson et al., 1999). Moreover, low crop load may negatively affect the net CO_2_ exchange rate of apple leaves (Palmer et al., 1997; Pallas et al., 2018). In “Braeburn”/M.26 trees, planted at 5 m × 2.5 m, mean mid-season leaf net CO_2_ exchange rate was reduced when the LA per fruit (LA:F) of the whole tree exceeded 830 cm^2^ (Palmer et al., 1997). In the present study, however, crop load did not have any effect on ^max^J_CO2_ (Figure 3), presumably because the LA:F, ranging from 340 cm^2^ to 780 cm^2^ (data not shown), did not exceed this threshold. Consequently, the reduction of photosynthetic performance can be assumed as a marginal influence on the present findings.

The feasibility of the FBC model was confirmed by comparing the modeled FBC of each individual tree and the measured mean fruit diameter of the trees as a reference diameter. The ratio obtained was close to 1, proving that the model meets the real-world conditions. Consequently, the FBC provides a concept for simulating the optimum crop load for each tree. The application of FBC for evaluating the actual crop load of each individual tree and addressing the precise management of orchards is potentially based on the decision of each individual tree.

Nevertheless, in the commercial orchard, crop load exceeded the estimated FBC in a considerable number of trees without any negative effects on the mean fruit diameter. It can be assumed that the model fails to account completely for the difference in canopy light extinction coefficient between the trees (Poblete-Echeverría et al., 2015). Actually, a few physiologically based tree metrics are available for tree design and annual pruning (Breen et al., 2021). Breen et al. (2021) reported that by means of the standardized six limbs per meter of vertical canopy height, light penetration into the inner parts of apple canopies can be increased without any negative consequences on light interception. This improves especially the percentage of a premium class fruit, and reduces variability among the fruit. In the present study, the number of limbs per meter of vertical canopy height varied between 6.6 and 21.3, exceeding the proposed ideal number.

The approach of modeling the LA demand to meet the carbon requirement of developing fruit to specific fruit sizes can provide an additional application. It may contribute to understand the effect of variable LA:F ratios on fruit mass, which was investigated on either whole trees of a similar size or exposed girdled branches (Palmer et al., 1997; Baïram et al., 2019). Remote sensing provides a new tool to study the LA demand per fruit for specific diameters in different planting systems. However, one limitation is that the LA demand is an average value of the whole tree, not representing the individual types of leaves and distances between leaves and fruit. Consequently, no conclusions about the fruit size distribution in the individual branch level can be made.

### Modeling the LA Demand for Different Fruit Sizes

To meet the consumer's preferences, commercial fruit quality requirements demand a minimum fruit diameter of 65 mm while at least 60% of the fruit surface must be covered with red blush. The firmness of a high-quality ‘Gala’ fruit should be below 62 N (Harker et al., 2008) and SSC at least 12% (Saei et al., 2011). In the present study, most of the fruit met these consumer preferences when the number of fruit per tree was in the range of the FBC estimated for the target fruit diameters (Figures 8, 10–12 and Supplementary Table 2). With the present approach, an assessment method for the optimum number of fruit per individual tree targeting a certain fruit size becomes available. For applying the FBC in a VRA or field-uniform thinning measure, a few variables are requested: The conversion factor for turning fruit diameter into FM can be obtained on the farm. The LA needs to be known, and here more methods and commercial services are becoming available at present (Tsoulias et al., 2021). For a field-uniform assessment, the mean LA of 5.5 m^2^, found in the present study can be applied as an example (Table 4). With target FM or diameter, known LA, fruit respiration rate from extension service or literature, and weather data from satellite or weather station, the calculation of FBC is enabled (Equation 12). The FBC for the desired fruit diameter can serve as the target crop load in thinning measures (Table 4), e.g., to evaluate whether and to what extent thinning practices are required. In order to account for the production system of the orchard, the TAPE can be considered additionally.

**Table 4:** FBC considering the four target fruit diameters (D) converted into fresh mass (FM) of ‘Gala’/M.9 apple trees in 2018 and 2019 of trees with a mean of 5.5 m^2^ total LA; the total absorbed photosynthetic energy (TAPE) (MJ fruit^−1^) per fruit considering the FBC; the measured FM receiving this TAPE per fruit, the ratio between the targeted fruit FM and measured FM.

**Year**	**D (mm), FM (g)**	**FBC (fruit tree^**−1**^)**	**TAPE fruit^**−1**^ (MJ fruit^**−1**^)**	**FM (TAPE fruit^**−1**^) (g)**	**Ratio between targeted FM and FM (TAPE fruit^**−1**^)**
2018	65, 102	130	5.4	116.8	0.9
	70, 129	98	7.1	126.0	1.0
	75, 161	79	8.8	135.3	1.2
	80, 198	66	10.5	144.5	1.4
2019	65, 102	139	5.5	141.0	0.7
	70, 129	105	7.3	159.6	0.8
	75, 161	85	9.0	178.1	0.9
	80, 198	71	10.8	196.7	1.0

A previous work indicated a lower firmness in apples grown on trees with high crop load compared to low crop load trees (Link, 2000; Serra et al., 2016). Therefore, it was expected that fruit firmness would respond to TAPE per fruit, as did FM and SSC. However, this was not found in the present study.

Both yield and average mass of fruit were directly affected by TAPE and TAPE per fruit confirming the validity of the concept of modeling the FBC of apple trees. With a similar approach, Wünsche et al. (1996) explained differences in productivity of apple growing systems by the amount of intercepted radiation capturing a 2-weeks period. Furthermore, a high TAPE per fruit (2018: 7.4 MJ fruit^−1^; 2019: 5.5 MJ fruit^−1^, Figure 11B) is required for the trees to achieve a high percentage of fruit with D > 65 mm. At this TAPE per fruit, representing a LA:F ratio of approx. 550 cm^2^, 80% of the apples reached D > 65 mm in both years. Thus, this LA:F can be seen as a threshold target for crop load management to achieve a marketable average fruit mass in the present orchard. The threshold is expected to differ in other orchards.

When the number of fruit per tree appeared in the range of FBC, the TAPE per fruit was above the 7.4 MJ fruit^−1^ only when targeting D > 70 mm in 2018; and above 5.5 MJ fruit^−1^ when targeting D > 65 mm in 2019 (Table 4). The modeled FBC slightly underestimated the actual FBC. However, with the presented empirical model (Figures 7, 8) a target fruit diameter for specific markets can be approached.

The SD of SSC in 2018 was negatively correlated with TAPE per fruit, indicating that differences in SSC can be reduced by the precise management of crop load. The maximum between-tree variability in the mean SSC was 1.4%, which was similar to the data previously reported for ‘Gala’ apples (Hoehn et al., 2003; Pilar Mata et al., 2006). Within-tree SSC is additionally influenced by the fruit position in the canopy (Nilsson and Gustavsson, 2007) and, thus, fruit exposure to sunlight (Zhang et al., 2016), distance to the leaves, and other sink organs. In ‘Gala’/M.26 apples, the mean SSC between fruit from the inner and outer part of canopies differed up to 1.4% (Feng et al., 2014).

The estimation of FBC of each individual tree can be applied to develop VRAs of thinning. Mechanical VRA in thinning based on the flower set of the trees avoided over-thinning of each individual tree with a low flower set, which could increase the fruit yield by 1.4–7.6 t ha^−1^ (Penzel et al., 2021). Knowledge on the actual FBC of each individual tree may prevent overestimation and underestimation of thinning intensity and yield as confirmed in two commercial apple orchards earlier. The number of fruit per tree of 23%, 31% of the considered trees were below the FBC, although the per tree flower cluster numbers would have enabled to meet the FBC (Penzel et al., 2020). Yield reduction due to the uniform thinning of trees with variable FBC may be avoided by the knowledge on FBC.

However, for a precise VRA in crop load management of each individual tree, FBC needs to be analyzed each year since the FBC of each individual tree differs between the years (Figure 9). Individual LA of trees of the fully developed canopies may be estimated from the early season LA or the number of spurs and extensions that shoots in a growth model of growing degree days (Lakso and Johnson, 1990). Furthermore, when the actual crop load data of each individual tree will become available (Apolo-Apolo et al., 2020; Tsoulias et al., 2020a), the difference between FBC and the actual crop load will provide a decision support for each individual tree, enabling VR thinning.

## Conclusion

The overall variability of LA per tree and the associated FBC were found in two consecutive years. This finding points to potentially erratic crop load management when field-uniform thinning intensity is applied.

The number of photons per fruit intercepted by the tree during the growing season determined fruit mass and SSC. To produce 80% of the fruit with a D > 65 mm, ≥7.4 MJ fruit^−1^ (2018), and ≥5.5 MJ fruit^−1^ (2019) were needed. Such values represented the LA to fruit ratio above 550 cm^2^ in the present orchard. The mean LA of 5.5 m^2^ provided the FBC ranging from 66 to 139 fruit when targeting varying harvest fruit diameters (65–80 mm). The corresponding TAPE per fruit ranged from 5.4 to 10.8 MJ fruit^−1^.

Consequently, the FBC to produce a desired mean fruit diameter per tree can be feasibly estimated based on the availability of LA data per tree. The branch autonomy considering source-to-sink and sink-to-sink distances needs to be further investigated, potentially by combining related models and advanced LiDAR readings distinguishing the type of leaf and fruit. With the carbon balance and new sensor data, the variable rate thinning adjusting the thinning intensity for each tree can, therefore, be supported.

## Data Availability Statement

The raw data supporting the conclusions of this article will be made available by the authors, without undue reservation.

## Author Contributions

MP: conceptualization, methodology, validation, formal analysis, writing—original draft, and writing—review and editing. WH: methodology, resources, and writing—review and editing. CW: writing—review and editing. NT: conceptualization, formal analysis, methodology, and writing. MZ-S: funding acquisition, methodology, writing—review and editing, and supervision. All authors contributed to the article and approved the submitted version.

## Conflict of Interest

The authors declare that the research was conducted in the absence of any commercial or financial relationships that could be construed as a potential conflict of interest.
