# Supplementary material for: Modeling of Individual Fruit-Bearing Capacity of Trees Is Aimed at Optimizing Fruit Quality of Malus x domestica Borkh. ‘Gala’
Source: Front Plant Sci. 2021 Jul 13;12:669909. doi: 10.3389/fpls.2021.669909 (PMC8315137; doi:10.3389/fpls.2021.669909)
Supplement: Supplementary file 1 [file Data_Sheet_1.pdf]

Supplementary material

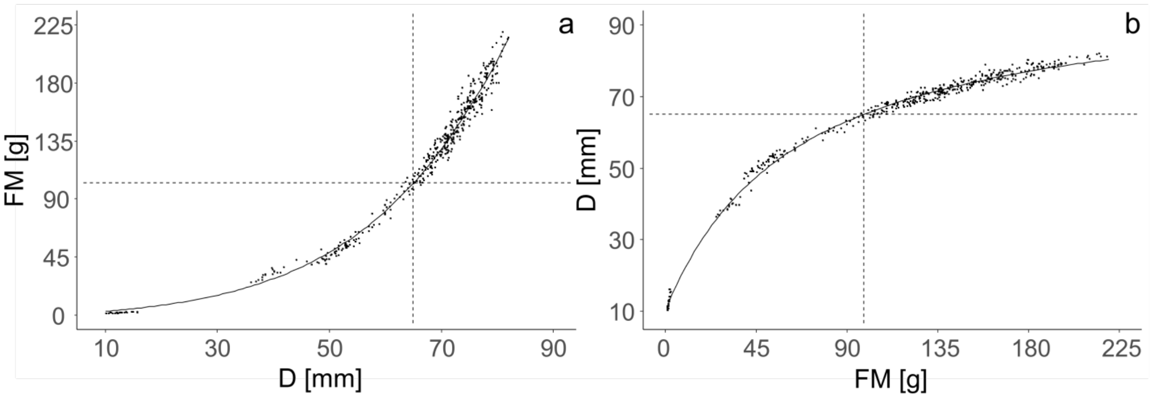

Figure S1

Allometric relationships between (a) diameter (D) and fresh mass (FM), (b) FM and D of developing ' Gala'/M.9 apple fruit (n = 460) sampled at several dates during the growing seasons in 2018 and 2019 (Fig. 2). The vertical lines indicates the minimum fruit diameter for market entry of 65 mm, the solid lines regression models to convert D into FM and FM into D. (FM = 939.73 - 199741/((1.05202)^{D} + 211.527), R^2 = 0.98; D = 10.465 + 90.45152 × FM × (64.614448 + FM)$^{-1}$, R^2 = 0.99)

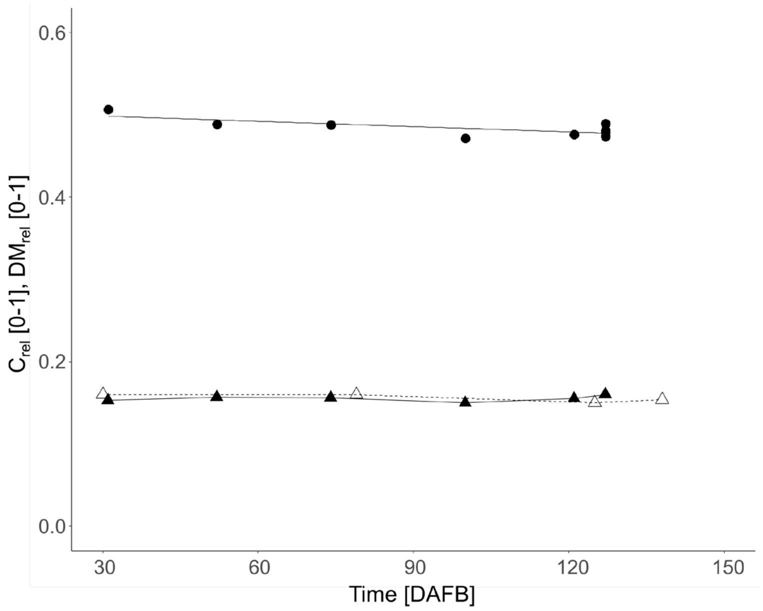

Figure S2

Time course in d after full bloom (DAFB) of average fraction of dry matter on fresh mass (triangles, DM_{rel}) in 2018 (triangle pointing up), 2019 (triangle pointing down) and fractions of elemental C on DM_{rel} in 2018 (circle, C_{rel}) of developing 'Brookfield Gala'/M.9 apple fruit. (C_{rel} = 50.837 - 0.0273 × DAFB, p=0.01, R^2 = 0.72)

Table S1

Normalised equations of seasonal fruit development considering fresh mass (FM_{norm}) and elemental C content (C_{norm}) of ' Gala' apple fruit expressed over time in days after full bloom (DAFB) in two years.

Year	Equations	No.
2018	$FM_{norm} = 1.16 - \frac{193.1}{(1.056)^{DAFB}+169.12}$	S1.1
	$C_{norm} = 1.28 - \frac{132.83}{(1.0476)^{DAFB} + 103.39}$	S2.1
2019	$FM_{norm} = 1.04 - \frac{323.99}{(1.068)^{DAFB}+ 312.55}$	S1.2
	$C_{norm} = 1.06223 - \frac{189.26}{(1.06)^{DAFB} + 177.63}$	S2.2

$C_{rel}(DAFB) = 50.837 - 0.0273 \times DAFB$, $p=0.01$, $R^2 = 0.72$ (Eq. S3)

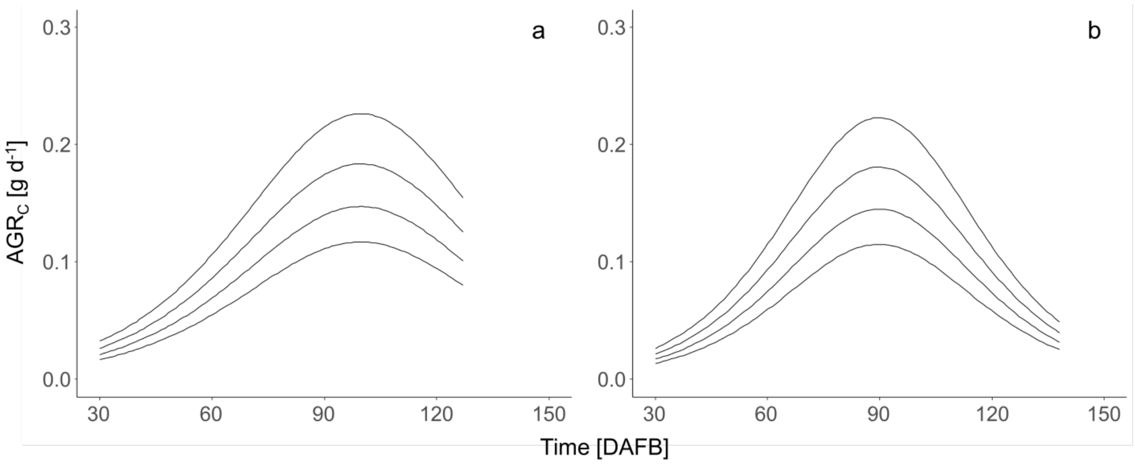

Figure S3

Absolute growth rate in C content (AGR_C) during 2018 (a) and 2019 (b) in the days after full bloom (DAFB) simulated for ' Gala'/M.9 apples with target fruit diameters of 65 mm, 70 mm, 75 mm and 80 mm (from the bottom to top).

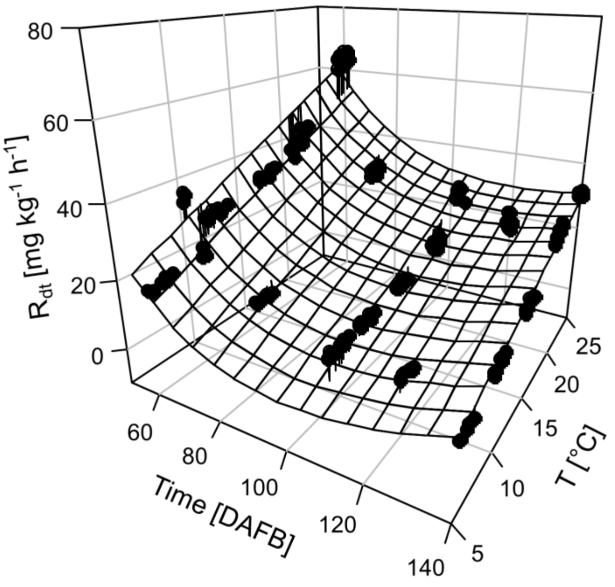

Figure S4

Fruit respiration rate, R_{dt}, of ' Gala' apple during cell expansion and ripening stage of fruit. The R_{dt} model considers the time, d after full bloom (DAFB), temperature (T): $R_{dt} = 425.6 - 122.7 \times \ln(DAFB) - 0.0097 \times DAFB \times T + 2.31 \times T + 1.31 \times DAFB$, $p<0.001$; $R^2 = 0.87$.

Table S2

Fruit firmness, hue angle of 'Gala' apple fruit and percentage of fruit with >60 % red blush of the fruit surface of 'Gala' trees in two years measured at harvest.

	F_{max} [N][1]			hue angle[1]			Percentage of fruit with > 60 % red blush [%][2]		
Year	Min.	Max.	Mean	Min.	Max.	Mean	Min.	Max.	Mean
2018	41	98	67	22	87	38	31	99	82
2019	50	115	86	-	-	-	44	100	64

[1]individual fruit, 2018: n=180, 2019: n=1240; [2]individual trees, 2018: n=100, 2019: n=70

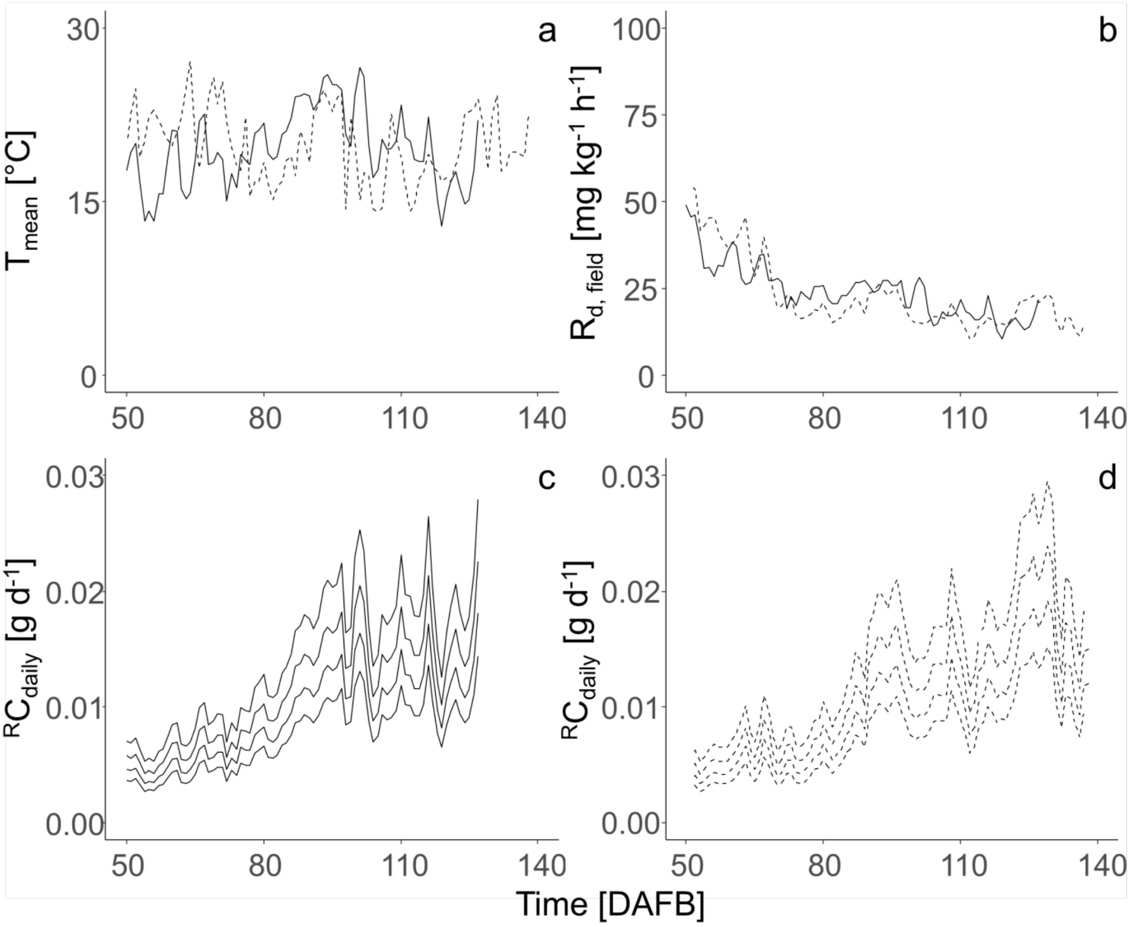

Figure S5

(a) Mean daily orchard temperature (T_{mean}) in 2 m height, (b) temperature (T_{mean})-corrected field-measured fruit respiration rate ($R_{d,field}$; 2018: solid line, 2019: dashed line), (c, d) amount of C respired per fruit and day ($^{R}C_{daily}$) of ' Gala'/M.9 apples with the target diameters of 65 mm, 70 mm, 75 mm and 80 mm (from the bottom up) during the days after full bloom (DAFB) in 2018 (c) and 2019 (d).
